# 4-(4-Fluoro­benzene­sulfonamido)­phenyl 4-fluoro­benzene­sulfonate

**DOI:** 10.1107/S1600536812009877

**Published:** 2012-03-17

**Authors:** Belal O. Al-Najjar, Tengku Sifzizul Tengku Muhammad, Habibah A. Wahab, Mohd Mustaqim Rosli, Hoong-Kun Fun

**Affiliations:** a Pharmaceutical Design and Simulation (PhDS) Laboratory, School of Pharmaceutical Sciences, Universiti Sains Malaysia, 11800 Minden, Pulau Pinang, Malaysia; b Malaysian Institute of Pharmaceuticals and Nutraceuticals, Ministry of Science, Technology and Innovation, SAINS@USM, No. 10, 11900 Persiaran Bukit Jambul, Pulau Pinang, Malaysia; c Department of Biological Sciences, Universiti Malaysia Terengganu, 21030 Kuala Terengganu, Terengganu, Malaysia; d X-ray Crystallography Unit, School of Physics, Universiti Sains Malaysia, 11800 USM, Penang, Malaysia

## Abstract

In the title compound, C_18_H_13_F_2_NO_5_S_2_, the complete mol­ecule is generated by a crystallographic inversion centre, and the O atom and the N—H group attached to the central ring are statistically disordered. The dihedral angle between the central and terminal benzene rings is 64.03 (6)°. In the crystal, N—H⋯O, C—H⋯F and C—H⋯O inter­actions link the mol­ecules into a three-dimensional network.

## Related literature

For a related structure showing similar statistical disorder of its O atom and NH group, see: Al Najjar *et al.* (2012 ▶). For background to the biological activity of benzene­sulfonates, see: Supuran *et al.* (2003 ▶). For the stability of the temperature controller used in the data collection, see: Cosier & Glazer (1986 ▶).
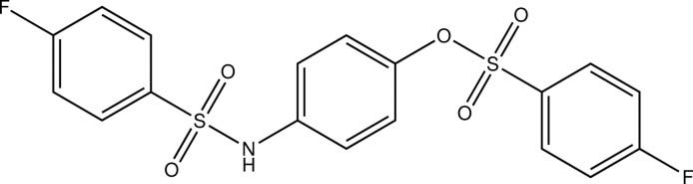


## Experimental

### 

#### Crystal data

C_18_H_13_F_2_NO_5_S_2_
*M* *_r_* = 425.41
Monoclinic, 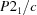

*a* = 8.9683 (1) Å
*b* = 11.0323 (1) Å
*c* = 9.3314 (1) Å
β = 102.363 (1)°
*V* = 901.85 (2) Å^3^
*Z* = 2
Mo *K*α radiation
μ = 0.35 mm^−1^
*T* = 100 K
0.37 × 0.33 × 0.21 mm

#### Data collection

Bruker SMART APEXII CCD diffractometer
Absorption correction: multi-scan (*SADABS*; Bruker, 2009 ▶) *T*_min_ = 0.883, *T*_max_ = 0.930
12757 measured reflections
3235 independent reflections
2940 reflections with *I* > 2σ(*I*)
*R*_int_ = 0.018

#### Refinement

*R*[*F* ^2^ > 2σ(*F* ^2^)] = 0.036
*wR*(*F* ^2^) = 0.100
*S* = 1.09
3235 reflections
127 parameters
H-atom parameters constrained
Δρ_max_ = 0.39 e Å^−3^
Δρ_min_ = −0.39 e Å^−3^

### 

Data collection: *APEX2* (Bruker, 2009 ▶); cell refinement: *SAINT* (Bruker, 2009 ▶); data reduction: *SAINT*; program(s) used to solve structure: *SHELXTL* (Sheldrick, 2008 ▶); program(s) used to refine structure: *SHELXTL*; molecular graphics: *SHELXTL*; software used to prepare material for publication: *SHELXTL* and *PLATON* (Spek, 2009 ▶).

## Supplementary Material

Crystal structure: contains datablock(s) I, global. DOI: 10.1107/S1600536812009877/hb6664sup1.cif

Structure factors: contains datablock(s) I. DOI: 10.1107/S1600536812009877/hb6664Isup2.hkl

Supplementary material file. DOI: 10.1107/S1600536812009877/hb6664Isup3.cml

Additional supplementary materials:  crystallographic information; 3D view; checkCIF report

## Figures and Tables

**Table 1:** Hydrogen-bond geometry (Å, °)

*D*—H⋯*A*	*D*—H	H⋯*A*	*D*⋯*A*	*D*—H⋯*A*
N1—H1*N*1⋯O2^i^	0.96	2.12	3.0630 (14)	169
C5—H5*A*⋯F1^ii^	0.95	2.37	3.2766 (16)	159
C6—H6*A*⋯O3^iii^	0.95	2.54	3.4130 (17)	152
C7—H7*A*⋯O2^iv^	0.95	2.60	3.3936 (17)	142

Symmetry codes: (i) 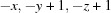; (ii) 
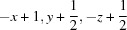
; (iii) 
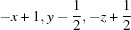
; (iv) 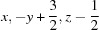.

**Fig. 1.**
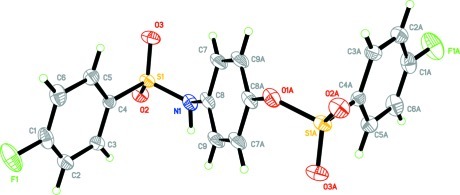
The first disorder component of the structure with 50% probability displacement ellipsoids. Hydrogen atoms are shown as spheres of arbitrary radius.

**Fig. 2.**
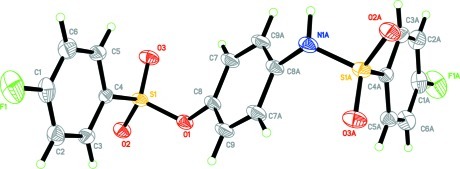
The second disorder component of the structure with 50% probability displacement ellipsoids. Hydrogen atoms are shown as spheres of arbitrary radius.

**Fig. 3.**
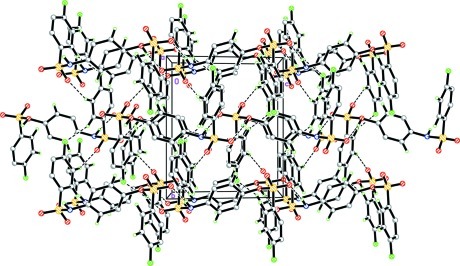
The crystal packing of (I). Dashed lines indicate hydrogen bonds. H atoms not involved in the hydrogen bond interactions have been omitted for clarity.

## supplementary crystallographic information

### Comment

As part of our ongoing structural investigations of benzenesulfonates (Al Najjar *et al.*, 2012) with potential biological activities (Supuran *et al.*, 2003), we now describe the synthesis and structure of the title compound, (I).

The asymmetric unit of the title compound consists of half the molecule with other half being generated by inversion centre. The O1 and N1 atoms occupy the same position to the central phenyl ring (Fig 1 and Fig 2), disordered with half occupancies each. A similar disordering is seen in a related structure with meta substituents on the terminal rings (Al Najjar *et al.*, 2012), although in this case, a crystallographic twofold axis generates the complete molecule. All parameters in (I) are within normal ranges. The dihedral angle between C1/C6 and C7—C9/C7A—C9A is 64.03 (6)° whereas the the C1/C6 ring and its symmetry equivalent C1A/C6A ring are constrained by symmetry to lie in a parallel orientation. In the crystal, N1—H1N1···O2^i^, C5—H5A···F1^ii^, C6—H6A···O3^iii^ and C7—H7A···O2^iv^ bonds (Table 1) link the molecules into a three-dimensional network (Fig. 3)

### Experimental

0.02 Mole of 4-fluorobenzenesulfonyl chloride was added to 0.01 mole of *p*-aminophenol dissolved in pyridine. Next, the reaction mixture was neutralized by adding hydrochloric acid. The precipitate formed was dissolved in 5% aqueous sodium hydroxide, and the sulfonamide recovered by adding 1:1 hydrochloric acid slowly. Re-crystallization of the product by slow evaporation of an ethyl acetate solution gave yellow blocks of (I).

### Refinement

N bound H atoms were located from a difference Fourier maps and refined using a riding model. The remaining H atoms were positioned geometrically and refined using a riding model with C—H = 0.95 Å and *U*_iso_(H) = 1.2*U*_eq_(C).

### Crystal data

**Table:**

C_18_H_13_F_2_NO_5_S_2_	*F*(000) = 436
*M**_r_* = 425.41	*D*_x_ = 1.567 Mg m^−^^3^
Monoclinic, *P*2_1_/*c*	Mo *K*α radiation, λ = 0.71073 Å
Hall symbol: -P 2ybc	Cell parameters from 7746 reflections
*a* = 8.9683 (1) Å	θ = 2.3–32.6°
*b* = 11.0323 (1) Å	µ = 0.35 mm^−^^1^
*c* = 9.3314 (1) Å	*T* = 100 K
β = 102.363 (1)°	Block, yellow
*V* = 901.85 (2) Å^3^	0.37 × 0.33 × 0.21 mm
*Z* = 2	

### Data collection

**Table:**

Bruker SMART APEXII CCD diffractometer	3235 independent reflections
Radiation source: fine-focus sealed tube	2940 reflections with *I* > 2σ(*I*)
Graphite monochromator	*R*_int_ = 0.018
φ and ω scans	θ_max_ = 32.6°, θ_min_ = 2.3°
Absorption correction: multi-scan (*SADABS*; Bruker, 2009)	*h* = −13→13
*T*_min_ = 0.883, *T*_max_ = 0.930	*k* = −16→16
12757 measured reflections	*l* = −12→14

### Refinement

**Table:**

Refinement on *F*^2^	Primary atom site location: structure-invariant direct methods
Least-squares matrix: full	Secondary atom site location: difference Fourier map
*R*[*F*^2^ > 2σ(*F*^2^)] = 0.036	Hydrogen site location: inferred from neighbouring sites
*wR*(*F*^2^) = 0.100	H-atom parameters constrained
*S* = 1.09	*w* = 1/[σ^2^(*F*_o_^2^) + (0.0442*P*)^2^ + 0.4073*P*] where *P* = (*F*_o_^2^ + 2*F*_c_^2^)/3
3235 reflections	(Δ/σ)_max_ < 0.001
127 parameters	Δρ_max_ = 0.39 e Å^−^^3^
0 restraints	Δρ_min_ = −0.39 e Å^−^^3^

### Special details

**Table:**

Experimental. The crystal was placed in the cold stream of an Oxford Cryosystems Cobra open-flow nitrogen cryostat (Cosier & Glazer, 1986) operating at 100.0 (1) K.
Geometry. All e.s.d.'s (except the e.s.d. in the dihedral angle between two l.s. planes) are estimated using the full covariance matrix. The cell e.s.d.'s are taken into account individually in the estimation of e.s.d.'s in distances, angles and torsion angles; correlations between e.s.d.'s in cell parameters are only used when they are defined by crystal symmetry. An approximate (isotropic) treatment of cell e.s.d.'s is used for estimating e.s.d.'s involving l.s. planes.
Refinement. Refinement of *F*^2^ against ALL reflections. The weighted *R*-factor *wR* and goodness of fit *S* are based on *F*^2^, conventional *R*-factors *R* are based on *F*, with *F* set to zero for negative *F*^2^. The threshold expression of *F*^2^ > σ(*F*^2^) is used only for calculating *R*-factors(gt) *etc*. and is not relevant to the choice of reflections for refinement. *R*-factors based on *F*^2^ are statistically about twice as large as those based on *F*, and *R*- factors based on ALL data will be even larger.

### Fractional atomic coordinates and isotropic or equivalent isotropic displacement parameters (Å^2^)

**Table:**

	*x*	*y*	*z*	*U*_iso_*/*U*_eq_	Occ. (<1)
S1	0.19643 (3)	0.57696 (3)	0.40553 (3)	0.02189 (8)	
F1	0.48038 (14)	0.10387 (9)	0.39858 (12)	0.0534 (3)	
O1	0.03218 (11)	0.56620 (9)	0.29564 (11)	0.0265 (2)	0.50
N1	0.03218 (11)	0.56620 (9)	0.29564 (11)	0.0265 (2)	0.50
H1N1	−0.0391	0.5177	0.3342	0.032*	0.50
O2	0.15869 (11)	0.59542 (9)	0.54583 (10)	0.02712 (18)	
O3	0.28500 (11)	0.66585 (8)	0.34966 (11)	0.0302 (2)	
C1	0.41366 (16)	0.21362 (12)	0.39786 (15)	0.0318 (3)	
C2	0.29275 (15)	0.22427 (12)	0.46723 (16)	0.0311 (3)	
H2A	0.2565	0.1562	0.5119	0.037*	
C3	0.22599 (13)	0.33732 (11)	0.46964 (14)	0.0269 (2)	
H3A	0.1428	0.3483	0.5166	0.032*	
C4	0.28240 (12)	0.43473 (10)	0.40228 (12)	0.01905 (19)	
C5	0.40364 (13)	0.42142 (11)	0.33233 (13)	0.0248 (2)	
H5A	0.4402	0.4889	0.2868	0.030*	
C6	0.47055 (16)	0.30807 (13)	0.32990 (15)	0.0323 (3)	
H6A	0.5533	0.2962	0.2826	0.039*	
C7	0.08657 (15)	0.60289 (12)	0.05220 (15)	0.0299 (3)	
H7A	0.1450	0.6725	0.0882	0.036*	
C8	0.02046 (14)	0.53190 (11)	0.14551 (13)	0.0258 (2)	
C9	−0.06539 (15)	0.43016 (12)	0.09444 (15)	0.0300 (3)	
H9A	−0.1097	0.3831	0.1597	0.036*	

### Atomic displacement parameters (Å^2^)

**Table:**

	*U*^11^	*U*^22^	*U*^33^	*U*^12^	*U*^13^	*U*^23^
S1	0.02541 (13)	0.01878 (14)	0.02456 (14)	0.00237 (9)	0.01219 (10)	0.00205 (9)
F1	0.0779 (7)	0.0299 (5)	0.0536 (6)	0.0271 (5)	0.0170 (5)	0.0026 (4)
O1	0.0272 (4)	0.0288 (5)	0.0277 (4)	0.0093 (3)	0.0154 (3)	0.0084 (4)
N1	0.0272 (4)	0.0288 (5)	0.0277 (4)	0.0093 (3)	0.0154 (3)	0.0084 (4)
O2	0.0327 (4)	0.0280 (4)	0.0236 (4)	0.0023 (3)	0.0127 (3)	−0.0018 (3)
O3	0.0369 (5)	0.0206 (4)	0.0376 (5)	−0.0023 (3)	0.0183 (4)	0.0035 (3)
C1	0.0406 (6)	0.0230 (6)	0.0289 (6)	0.0110 (5)	0.0008 (5)	−0.0027 (5)
C2	0.0334 (6)	0.0211 (5)	0.0364 (6)	−0.0012 (4)	0.0020 (5)	0.0050 (5)
C3	0.0237 (5)	0.0239 (5)	0.0336 (6)	0.0002 (4)	0.0073 (4)	0.0065 (5)
C4	0.0185 (4)	0.0189 (5)	0.0197 (4)	0.0005 (3)	0.0042 (3)	−0.0003 (4)
C5	0.0237 (5)	0.0267 (6)	0.0259 (5)	0.0030 (4)	0.0096 (4)	0.0008 (4)
C6	0.0342 (6)	0.0342 (7)	0.0306 (6)	0.0130 (5)	0.0113 (5)	−0.0003 (5)
C7	0.0329 (6)	0.0269 (6)	0.0349 (6)	0.0075 (5)	0.0179 (5)	0.0146 (5)
C8	0.0281 (5)	0.0255 (5)	0.0281 (5)	0.0116 (4)	0.0159 (4)	0.0120 (4)
C9	0.0313 (6)	0.0289 (6)	0.0351 (6)	0.0076 (4)	0.0188 (5)	0.0162 (5)

### Geometric parameters (Å, º)

**Table:**

S1—O3	1.4289 (9)	C3—H3A	0.9500
S1—O2	1.4351 (9)	C4—C5	1.3905 (15)
S1—O1	1.6089 (11)	C5—C6	1.3893 (17)
S1—C4	1.7515 (11)	C5—H5A	0.9500
F1—C1	1.3499 (15)	C6—H6A	0.9500
O1—C8	1.4332 (16)	C7—C9^i^	1.389 (2)
O1—H1N1	0.9606	C7—C8	1.3943 (16)
C1—C6	1.374 (2)	C7—H7A	0.9500
C1—C2	1.382 (2)	C8—C9	1.387 (2)
C2—C3	1.3858 (18)	C9—C7^i^	1.389 (2)
C2—H2A	0.9500	C9—H9A	0.9500
C3—C4	1.3936 (16)		
			
O3—S1—O2	119.57 (6)	C5—C4—C3	121.74 (11)
O3—S1—O1	108.83 (6)	C5—C4—S1	119.61 (9)
O2—S1—O1	103.25 (5)	C3—C4—S1	118.64 (8)
O3—S1—C4	109.11 (5)	C6—C5—C4	119.12 (12)
O2—S1—C4	109.49 (5)	C6—C5—H5A	120.4
O1—S1—C4	105.62 (5)	C4—C5—H5A	120.4
C8—O1—S1	120.53 (7)	C1—C6—C5	118.09 (12)
C8—O1—H1N1	107.7	C1—C6—H6A	121.0
S1—O1—H1N1	113.2	C5—C6—H6A	121.0
F1—C1—C6	118.34 (13)	C9^i^—C7—C8	118.74 (13)
F1—C1—C2	117.73 (13)	C9^i^—C7—H7A	120.6
C6—C1—C2	123.93 (12)	C8—C7—H7A	120.6
C1—C2—C3	117.99 (12)	C9—C8—C7	121.28 (12)
C1—C2—H2A	121.0	C9—C8—O1	117.95 (10)
C3—C2—H2A	121.0	C7—C8—O1	120.67 (13)
C2—C3—C4	119.13 (11)	C8—C9—C7^i^	119.98 (11)
C2—C3—H3A	120.4	C8—C9—H9A	120.0
C4—C3—H3A	120.4	C7^i^—C9—H9A	120.0
			
O3—S1—O1—C8	59.87 (10)	O1—S1—C4—C3	−71.39 (10)
O2—S1—O1—C8	−172.10 (9)	C3—C4—C5—C6	−0.18 (18)
C4—S1—O1—C8	−57.16 (10)	S1—C4—C5—C6	−179.74 (10)
F1—C1—C2—C3	178.60 (12)	F1—C1—C6—C5	−178.59 (12)
C6—C1—C2—C3	−0.7 (2)	C2—C1—C6—C5	0.7 (2)
C1—C2—C3—C4	0.23 (19)	C4—C5—C6—C1	−0.25 (19)
C2—C3—C4—C5	0.18 (18)	C9^i^—C7—C8—C9	−0.15 (19)
C2—C3—C4—S1	179.75 (10)	C9^i^—C7—C8—O1	−176.54 (11)
O3—S1—C4—C5	−8.66 (11)	S1—O1—C8—C9	122.24 (11)
O2—S1—C4—C5	−141.24 (9)	S1—O1—C8—C7	−61.25 (13)
O1—S1—C4—C5	108.18 (10)	C7—C8—C9—C7^i^	0.2 (2)
O3—S1—C4—C3	171.77 (9)	O1—C8—C9—C7^i^	176.64 (11)
O2—S1—C4—C3	39.18 (11)		

Symmetry code: (i) −*x*, −*y*+1, −*z*.

### Hydrogen-bond geometry (Å, º)

**Table:**

*D*—H···*A*	*D*—H	H···*A*	*D*···*A*	*D*—H···*A*
N1—H1*N*1···O2^ii^	0.96	2.12	3.0630 (14)	169
C5—H5*A*···F1^iii^	0.95	2.37	3.2766 (16)	159
C6—H6*A*···O3^iv^	0.95	2.54	3.4130 (17)	152
C7—H7*A*···O2^v^	0.95	2.60	3.3936 (17)	142

Symmetry codes: (ii) −*x*, −*y*+1, −*z*+1; (iii) −*x*+1, *y*+1/2, −*z*+1/2; (iv) −*x*+1, *y*−1/2, −*z*+1/2; (v) *x*, −*y*+3/2, *z*−1/2.
